# The semantic storage loss score: An Algorithm for measuring an individual's level of semantic storage loss due to temporal lobe damage in neurodegenerative disease

**DOI:** 10.1371/journal.pone.0235810

**Published:** 2020-08-18

**Authors:** Carlos Roncero, Jim Nikelski, Stephan Probst, Alita Fernandez, Alex Thiel, Howard Chertkow

**Affiliations:** 1 Lady Davis Institute for Medical Research, Jewish General Hospital, McGill University, Montréal, Québec, Canada; 2 Baycrest Health Sciences, Rotman Research Institute, Toronto, Ontario, Canada; 3 Nuclear Medicine, Jewish General Hospital, McGill University, Montréal, Québec, Canada; University of Ljubljana, SLOVENIA

## Abstract

Anomia is common in Primary Progressive Aphasia (PPA), and there is considerable evidence that semantic problems (as opposed to impaired access to output word phonology) exist in many PPA individuals irrespective of their strict subtype, including a loss of representations from semantic memory, which is typical for people with the semantic variant of PPA. In this manuscript we present a straightforward novel clinical algorithm that quantifies this degree of semantic storage impairment. We sought to produce an algorithm by employing tasks that would measure key elements of semantic storage loss: a) whether an unrecalled name could be retrieved with cues; b) if performance for items was consistent across tasks; and c) the degree to which a participant’s performance was related to general severity of cognitive impairment rather than semantic loss. More specifically, these tasks were given to 28 individuals with PPA (12 participants had a clinical diagnosis of atypical Alzheimer’s Disease with the logopenic variant of PPA; the remaining 16 participants received a clinical diagnosis of Frontotemporal dementia (11 were classified as the non-fluent variant of PPA and five were the semantic variant of PPA). Scores from these tasks produced a single omnibus semantic memory storage loss score (SSL score) for each person that ranged from 0.0 to 1.0, with scores closer to 0 more indicative of semantic storage loss. Indeed, supporting the hypothesis that our scores measure the degree of semantic storage loss, we found participants with the semantic variant of PPA had the lowest scores, and SSL scores could predict the degree of hypometabolism in the anterior temporal lobe; even when only people with the logopenic variant of PPA were examined. Thus, these scores show promise quantitating the degree of a person’s semantic representation loss.

## Introduction

Individuals with acquired brain damage to the left hemisphere often demonstrate the inability to name pictures (anomia). Once visual processing problems have been excluded (by testing of the visual system), the locus of the deficit generally lies in systems and networks involved in language processing. In many individuals with stroke-induced Broca’s aphasia (or non-fluent Primary Progressive Aphasia), the problem lies not with knowledge of concepts, but arises only at the output lexical level, wherein the sound forms of words are contacted [1, 2]. In another set of individuals with anomia from stroke or neurodegenerative disease, the locus of impairment producing anomia lies at the level of semantic cognition [3, 4]. Consistent with this argument, a large body of work has coalesced around the conceptual framework of Controlled Semantic Cognition (CSC), which posits the existence of two cortical networks. One network, largely focused around an anterior temporal lobe (ATL) hub, is concerned with storage of semantic concepts. A second network, largely involving the left inferior frontal lobe and the left posterior temporal-parietal lobe cortex, comes into play for controlled semantic processing, be it disambiguating words, assigning alternative meanings, or the carrying out of executive control functions for semantic search [5–11]. This latter network is highly reminiscent of earlier concepts regarding “semantic access disorders” [8, 12–15].

Supporting the argument that two cortical networks exist, one concerned with the storage of semantic concepts versus one concerned with semantic processing; studies have examined participants with brain damage to the ATL, associated with semantic dementia and the storage of concepts, versus people described as having semantic aphasia, with anomia and brain damage external to the ATL. Brain damage from stroke producing semantic aphasia appears to manifest impaired function of the controlled semantic processing network. Semantic aphasia patients are individuals with stroke producing multi-modal semantic impairment, usually resulting from damage involving the left prefrontal cortex or regions in and around the temporo-parietal area. They show semantic (and not purely phonological) deficits, which may be evidenced in the non-verbal as well as the verbal domains and involve loss of comprehension of concepts as indicated by tests of associative matching [7, 16]. These deficits appear to largely affect executive control functions for semantic search and are inconsistent between tasks and over time–a concept may be available for output yet lost on comprehension tasks. Furthermore, they also manifest evidence of executive function deficits on neuropsychological testing. In contrast, certain individuals with Neurodegenerative diseases (NDD) produce a cognitive syndrome of Primary Progressive Aphasia (PPA), which can often manifest what appears to be a specific disruption of the semantic storage network. Such individuals are (most often) clinically labelled as the semantic variant of PPA (svPPA), although other forms of PPA can also manifest semantic disruption [17–20]. Group studies of svPPA suggest that these individuals have actual semantic representation loss (termed “semantic storage disorders”; [13, 21, 22]). The same studies suggest that such patients can have intact or relatively good executive function, and the poor naming ability is less related to executive function. Additionally, in most anatomical studies, there is demonstrable damage to the ATLs in svPPA. In short, the different symptom clusters suggest that different language networks (controlled semantic processing vs. semantic storage) are implicated.

Two other distinguishing characteristics have been enumerated as separating semantic processing from semantic storage disorders. Firstly, cueing, with phonemic or semantic cues, has been noted as a characteristic that distinguishes storage from processing disorders [12, 23]. Individuals with semantic processing deficits achieve normal-like performance levels if given cues for the names of concrete objects; semantic storage deficits are characterized by severe anomia which is not improved by cues. Secondly, consistency of response between comprehension and production tasks is another distinguishing feature. In storage deficits, an object-specific deficit is demonstrable during comprehension tasks (e.g., pointing to the correct picture in response to a verbal label) in addition to a deficit in producing the names themselves. In semantic processing disorders, the comprehension deficit may occur for different items, or not at all, but they do not consistently fail across tasks on the same conceptual items [7, 24].

Obviously, in a normal healthy brain, both semantic processing and semantic storage networks are active and present, but at the level of semantic cognition in a neurodegenerative disease, anomia can reflect impairment in one or both networks. The research relevant to this manuscript has involved participants with stroke or progressive neurological diseases who are typically asked to complete naming tasks where images of concreate objects are presented, as previously cited. For this reason, a major component of CSC is explaining why the different groups show different symptoms profiles despite both displaying anomia. Briefly, as stated by Jefferies and Lambon Ralph [7], these distinct symptom profiles are argued to reflect two interacting principal components: (i) a set of amodal representations and (ii) executive processes that help to direct and control semantic activation in a task-appropriate fashion. These amodal semantic representations are argued to exist within a semantic hub within the ATL and are considered amodal because they lack any inherent semantics and instead receive contextually relevant semantic information from association cortices (verbal and non-verbal); brain regions considered semantic control areas, such as the posterior temporal lobe. For this reason, when damage occurs in the ATL, it is predicted to impact all aspects of semantics related to a concept because the afferent and efferent neural connections that typically retrieve the relevant semantic information are damaged. Consistent with the argument that damage to the ATL should impact all aspects of semantics related to a specific category, participants with semantic dementia are found to do poorly on semantic tasks, regardless of modality, as well as responding poorly to cues, in addition to anomia, despite often showing relatively good general cognition [9]. The loss of these amodal representations within the ATL is considered a semantic *storage* impairment, reflecting a *semantic storage loss*. In turn, the algorithm presented here aims to quantify the degree of semantic storage loss present in an individual demonstrating anomia by indirectly testing the degree of amodal representation loss in the ATL.

### Which primary progressive aphasia subgroups show semantic deficits?

The argument that one brain network is more related to semantic processing processes, whereas another network is more devoted to semantic storage, has implications when discussing different forms of PPA. In the cognitive syndrome of PPA, there is insidious aphasia almost always related to left perisylvian cortical degeneration. This aphasia is initially the most salient cognitive impairment, and gradually progresses to generalized dementia [25, 26]. The underlying pathology is variable, but it is generally an NDD with the underlying brain pathology of Alzheimer’s Disease (AD) or Frontotemporal dementia (FTD), associated with an accumulation of tau protein or TDP43. A consensus conference chaired by Dr. Gorno-Tempini in 2011 [27] drew up criteria for the distinct profiles recognized in many cases of PPA, including their core features along with ancillary findings. These are now designated as the non-fluent variant of PPA (nfvPPA), reflecting prominent left frontal involvement, the semantic variant of PPA (svPPA), reflecting primary ATL involvement, or the logopenic variant of PPA (lvPPA), reflecting posterior left temporo-parietal involvement [27].

Virtually all recent PPA psychological studies have involved group comparisons utilizing these PPA profiles, which has unfortunately led to a common pitfall of categorization; namely a tendency to exaggerate group differences, while minimizing or ignoring the heterogeneity within each group. For example, many individuals with PPA do not fit neatly into these strict categories and are referred to as “mixed” [28–33]. All of these individuals (and indeed, almost all people with aphasia), regardless of PPA type, demonstrate anomia, the inability to name objects. Indeed, while Mesulam described three distinct core clinical profiles [17, 25, 26], it was also noted that there were overlap mixed cases of PPA with characteristics of more than one profile. Furthermore, many of the brain areas impacted by PPA affect the brain areas believed to be involved in the controlled semantic processing and semantic storage networks. For example, as previously noted, anomia can arise due to problems in lexical access and retrieving the phonological word form for objects. The latter problem is sometimes viewed as a problem related to the frontal lobe, and often found in individuals with nfvPPA who indeed have left frontal lobe pathology [30, 34]. However, the anomia can also be due to a semantic level problem, but one consisting of impaired semantic access, or semantic executive control, also related to left frontal lobe pathology [25, 30, 34–36]. In contrast, criteria for svPPA includes impaired object naming and single word comprehension, along with impaired object knowledge for low familiarity items [27]. Semantic impairment with profound anomia is considered most characteristic of svPPA cases. In summary, while group differences are generally correct, semantic loss is found in many PPA individuals, even those who do not fit the pattern of svPPA, including individuals that show the core features characteristic of lvPPA, nfPPA, and mixed PPA groups [25, 35, 36].

In-depth studies of PPA participants have noted semantic impairment across all diagnostic subcategories of PPA [17–20]. The 2011 consensus criteria [27] specified that semantic loss was only a core characteristic of svPPA, and lack of semantic problems constitutes “ancillary” criteria for the other two syndromes. This is problematic for many researchers, reflects a pitfall of categorization, and the rigidity of the criteria have been critiqued [17]. While the Gorno-Tempini criteria for nfvPPA [27] include core features along with relatively spared single word comprehension and spared object knowledge, 10% of nfvPPA in fact show impaired single word comprehension [20]. In our clinical experience, many PPA cases lacking the ancillary criteria are classified as nfvPPA rather than mixed, and the border zone of the diagnostic subtypes of PPA remain fuzzy at best [25, 35, 36]. A recent paper found that few individuals meet all the criteria for lvPPA, but most of those classified as mixed PPA have imaging abnormalities identical to those with lvPPA, suggesting that all share a focal but heterogeneous form of Alzheimer Disease pathology [37]. Regarding lvPPA, Grossman notes (2010) that “As the condition of patients (with lvPPA) worsens… difficulty in word comprehension can develop…The features of lvPPA are reminiscent of the language impairments frequently described in AD. Such features include naming difficulty…that can progress to impaired lexical comprehension.” [29].

In summary, it is not surprising that frontal and parietal atrophy can affect semantic processing, since the neural substrate of semantic processing includes areas in parieto-temporal (the locus of lvPPA), the left frontal lobe (the locus of nfvPPA), as well as temporal lobe and temporal pole (the usual locus of lvPPA cases). When semantic deficits are responsible for the anomia in nfvPPA and lvPPA, the pattern of impairment tends to reflect preserved but inaccessible semantic representations of concrete objects, referred to above as a deficit in the “semantic processing” network. We will follow in the footsteps of these other researchers who are “relaxing” the overly stringent Gorno-Tempini criteria by downplaying ancillary features and using the terminology to indicate core features only. Thus, hereafter, we will refer to groups of individuals roughly classified as syndromes of nfvPPA, svPPA, and lvPPA, despite the presence of semantic deficits across all the PPA individuals studied.

### Moving from groups to individuals in PPA assessment

While the cognitive syndromes and the brain-behavioural relationships described above occur in individuals, most of the clinical/imaging/pathology evidence for these syndromes has rested on group studies. This paradigm requires groups be well matched, yet distinct, since statistical power for finding group differences is inherently more powerful when inter-group variability is greater than intra-group variability. Therefore, group PPA studies will tend to restrict the possible variability to produce a homogenous group that may improperly reflect the range of deficits found in each PPA subgroup because individuals who fail to adhere to strict classification criteria may be simply omitted from research. In turn, results found may only be valid for a particular sub-group within a specific PPA sub-type, and only when the individual shares the same characteristics as the group examined. In truth, however, different characteristics may be at play within the same diagnostic group. Corbett, Jefferies, Burns, and Lambon-Ralph [21], for example, have argued that progressive damage in the ATL in individuals with typical AD could result in affected individuals transitioning from having semantic processing deficits to demonstrating additional semantic storage deficits. In their study, Corbett and colleagues had classified all participants scoring 14 or lower on the MMSE as being more severe than those scoring above 19 and predicted the lower scoring individuals would present more symptoms consistent with a semantic storage deficit. Results were consistent with this prediction and shows how within a single diagnostic group (AD) there can be individuals who display a semantic processing deficit or elements of both a processing and storage deficit. However, aside from this report, virtually all previous studies [7, 38–43] have established neuropsychological profiles for semantic storage and semantic processing disorders on a group basis but failed to develop a method whereby these can be practically operationalized for any individual to aid in diagnosis. In other words, although individuals can be classified as having a particular PPA sub-type, we lack the means to differentiate individuals belonging to the same PPA variant beyond discussing severity levels. These gross classification schemes allow only for vague classifications at the individual level. Based on Corbett’s results, for example, one is left to conclude that anyone scoring below 14 on the MMSE will likely present a semantic storage impairment, but there is no information regarding the degree of semantic storage impairment in that individual. In other words, inter-group differences are overemphasized while intra-group differences are diminished. Also, while MMSE scores may suggest the degree of overall dementia severity in people with AD, the task itself measures impairment across a range of cognitive domains separate from the degree of semantic storage loss. Therefore, it is also possible for an individual to score poorly on the MMSE due to poor general cognitive skills, but theoretically have an intact semantic system if areas such as the anterior temporal lobes are relatively spared. In summary, the MMSE can do a poor job of quantifying the degree of semantic storage loss in an individual.

Tests of semantic memory exist, particularly, the Camels and Cactuses test [44], but all of these tasks have been designed to distinguish impaired semantic memory versus normal semantic memory. Considering the observed variability among people diagnosed with PPA, it would be advantageous to be able to assess individuals on a set of key language tasks that would allow for the formation of a PPA profile. As argued by Lambon Ralph and colleagues for people with post-stroke aphasia [45–49], as well as individuals with PPA [9], there should be an allowance for graded differences as multiple neurocognitive systems may be implicated, as opposed to a single underlying neural system. In order to develop a more complete profile of a person, Individuals can have their performance measured in areas such as syntax, articulation, naming, and semantic memory, with each skill also reflecting corresponding brain areas [50–54]. Considering the traditional and current classification system, nfvPPA would still be people whose primary symptom is articulation impairment and agrammatism, and svPPA individuals would likely still demonstrate semantic storage deficits as a primary symptom. A system based on individual profiles for multiple forms of impairment would allow for graded differences both among the different PPA sub-types, and within the PPA sub-groups, which would undermine the pitfalls of categorization. Such scores would also allow for a set of non-group studies and related analyses that allow for greater variability as the need for homogenous groups would be removed.

In this paper, we will tackle the need to operationalize the degree of semantic storage impairment in an individual. More specifically, we will demonstrate how the administration of a small set of tests can be used to create a semantic storage loss score (SSL Score) for each participant with anomia. Measuring the degree of an individual’s semantic storage impairment is important because it a) strongly suggests svPPA when severe, b) can be found across PPA sub-types [27], and c) can evaluate the level of progression of semantic storage loss for people with lvPPA, as discussed by Corbett and colleagues [5]. Measuring the degree of a semantic storage deficit individually also allows predictions based on an individual’s performance and allows for the comparison of participants within a deficit type (e.g., mild vs. severe semantic storage deficit), where related symptoms could differ.

### Measuring semantic storage loss

We will discuss our general approach to test construction generalities here, and reserve specific details for the methods section. The semantic storage loss score was constructed using three scores derived from four tasks that are averaged to obtain an omnibus score. To derive the first two scores, participants are given an initial naming task to obtain a baseline list of hits and misses. The naming tasks (spontaneous and cued naming) focused on concrete imageable item concepts, for which there is far greater literature and consistent evidence of loss in brain damage. For missed items, participants are given a phonemic cue (the first two phonemes of the object label), and the proportion of missed items recalled with a cue is noted. In this manner, the first score in the semantic storage loss algorithm is produced: the proportion of missed items recalled with a cue. Previously, we noted that people with semantic storage deficits fail to improve greatly when given cues; thus, people with severe semantic storage deficits are predicted to obtain a low score.

Next, participants were presented with an array of images that represented a semantic category (e.g., domestic animals), given a target word orally, and were asked to point to the image corresponding to that word. The proportion of items initially missed in the Naming task, as well as when given a cue, but correctly identified in this pointing task, was noted. In this manner, the second score in the semantic storage loss algorithm is calculated: the proportion of items initially missed even with a cue that were correctly identified in the pointing task. Failing to correctly identify the object, despite the simpler task and change of modality, would be considered a miss, leading to scores closer to zero and suggestive of a semantic storage deficit as the same object concept would be affected for a comprehension task [55]. In summary, both proportion scores (ranging from 0.0 to 1.0) were designed to measure different aspects of semantic storage deficits, with lower scores more suggestive of a semantic storage deficit as it would indicate more misses for both cueing and pointing. For example, a participant who failed to spontaneously name an object, but could do so when given a cue, would gain a point for doing so and ultimately obtain a higher score than someone who failed to recall the object name when given a cue, but was able to do so in the matching task, who in turn would score higher than participants who failed even on the matching task.

The fourth task given, to produce the third score of the algorithm, was the Mini-Mental State Exam, or MMSE [56]. Past studies comparing groups with semantic processing impairment versus those with semantic storage impairment have noted that individuals can have severe semantic storage deficits alongside a relative absence of cognitive impairment in other domains [57]. In contrast, individuals with semantic processing problems generally show impairment of general cognition which would be reflected in a lower MMSE score. Thus, we wished to derive a measure to use as a rough indicator of overall cognitive function and we settled on the MMSE as an adequate measure. Despite its shortcomings, the MMSE has proven a durable screen for severity of dementia and continues to be commonly used by clinicians. We recognize that language comprehension and output are a component of the MMSE; there are three basic common items for naming and the instructions are all given verbally. However, most of the instructions used are simple questions, and even people with moderate dementia remain able to comprehend and respond appropriately to all the other questions even in the face of semantic memory impairment as seen in svPPA. Verbal working memory enters into instructions for carrying out the three-step command and serial seven subtractions, but impairment in these domains is not the same as semantic memory loss [58–59]. There are tests of attention, working memory, and episodic memory and orientation, giving a broad range of cognitive abilities and allowing us an acceptable measure of overall cognitive severity. We found this conceptually preferable to using an entirely non-verbal task such as Ravens Matrices. Consistent with previous studies, it was predicted that a semantic storage deficit was likely when a person had a poor naming score, but scored much better on a task of general cognitive function, such as the MMSE. For this reason, the algorithm was designed to capture the possible presence of poor naming despite good general cognition. With this assumption in mind, for each participant, we calculated the proportion score for the Naming task, as well as their proportion score for the MMSE, and placed both into a ratio between a person’s naming proportion score and sum of their MMSE and Naming proportion scores (Naming Proportion Score / (Naming Proportion Score + MMSE Proportion Score)). In this manner, the equation has the following structure:

$$\text{Domain:}\;\begin{array}{c} x\;\left(\text{Naming}\;\text{Proportion}\;\text{Score},\;0.0–1.0\right)\\ y\;\left(\text{MMSE}\;\text{Proportion}\;\text{Score},\;0.0–1.0\right) \end{array}$$

Function:f(x,y)=x/(x+y)

Because the range of X and Y are the same, the equation gives X and Y equal weight, and will be 0.5 when X = Y, but X + Y ≠ 0. Meanwhile, the range of possible outcomes is between 0.0 and 1.0, and the equation will increasingly approach 1.0 the greater X is larger than Y, but 0, the greater Y is larger than X. Returning to the tasks used, the equation’s structure in turn means the value will approach 0 when a person’s naming proportion score is inferior to their MMSE proportion score (consistent with storage deficits), but will approach 1 when the MMSE proportion score is worse than their naming proportion score, suggesting the anomia may be due more to general cognitive impairment than semantic storage loss. In other words, when poor naming ability occurs alongside relatively preserved general cognitive function, anomia is probably due more to semantic storage deficits, and this third score was designed to catch this variable.

**Fig 1:**
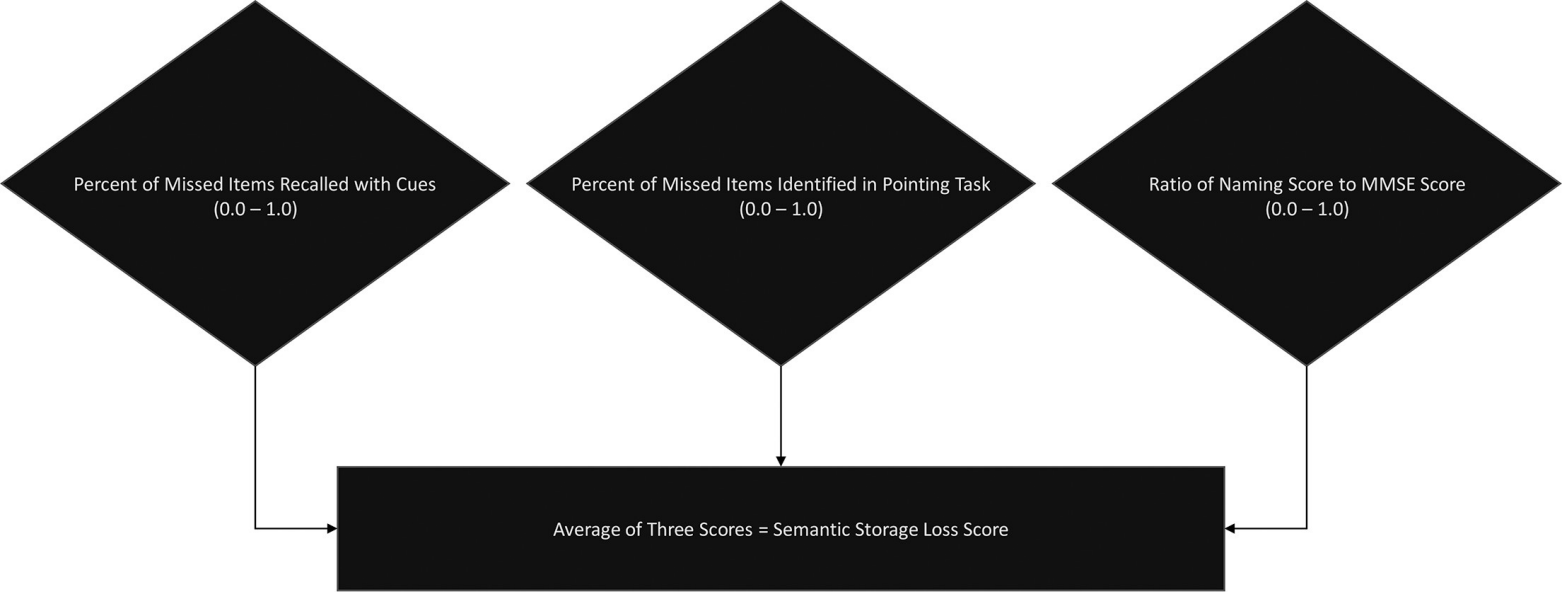
Semantic storage loss calculation.

In summary, we calculated one score to measure the cueing effect, one score to measure response consistency, and one measure of the ratio of naming to general cognition, where all three scores ranged from 0.0 to 1.0, and scores in each case suggested a more severe storage deficit when approaching zero. We had no prior bias regarding which individual measure was most important for predicting the degree of a semantic storage deficit; thus, due to the similarity of range and direction of each measure, we averaged the three scores to give each an equal weight in the final omnibus semantic storage loss (SSL) score, as displayed in Fig 1.

Consistent with the individual measures, lower SSL scores approaching 0 reflect more concrete object loss from semantic memory. Using all three scores also increases the specificity of the measure. While a single score alone (e.g. proportion of missed items recalled with a cue) may be somewhat informative for indicating an impairment for either semantic processing or storage, it would likely be less sensitive to graded differences between participants. For example, participants who score well on the MMSE, despite severe anomia, could be identified as probably being svPPA, but assessing this score alone would leave less clear those individuals who score poorly on the MMSE alongside naming impairment.

### Correlating semantic storage loss scores with anterior temporal lobe hypometabolism

As previously noted, damage in the ATL is associated with semantic storage deficits [60]. In turn, an important test of our SSL scores as a shorthand indicator of concrete object representation loss should be verifying that they can also predict the degree of ATL damage in an individual. To check for this prediction, we assessed the correlations between SSL scores and hypometabolism levels in the ATL, as well as the dorsolateral prefrontal cortex (dlpFC), established via FDG PET imaging that was carried out for all participants, to check that lower SSL scores correlated with a greater degree of temporal pole hypometabolism irrespective of diagnosis. All PPA participants received a FDG-PET and MRI no later than three months after their SSL score was calculated. A significant correlation with the ATL would add construct validity and indicate that our scores were correctly assessing participants’ level of impairment. Lack of correlations with hypometabolism in the dlpFC would constitute evidence that SSL scores were unrelated to brain regions involved in executive function or general disease severity. After running this analysis for all enrolled PPA participants, it would be run again only for people with lvPPA (generally an atypical form of AD). As noted above, people with lvPPA, as they progress, are expected to have a heterogeneous degree of concrete object representation loss, reflecting their respective degree of ATL damage. In turn, some people within this sub-type may present a semantic processing deficit, while others will also present concrete object representation loss (i.e., a loss of amodal representations). If SSL scores can correlate with the degree of ATL hypometabolism within this group, it would be additional strong evidence for its construct validity. Thus, these groups serve as a good test for SSL scores’ sensitivity to inter-group differences that should be related to a person’s respective ATL damage. Also, a significant correlation would provide strong evidence that increasing levels of a semantic storage deficit are causally related to ATL hypometabolism.

We also wanted to verify our previous argument that the average of the three individual scores of the SSL was a stronger predictor than each of these scores alone. It is possible that one to two of the individual components of the SSL, or the Cambridge Camels and Cactus Picture Task (a standard test of semantics, CCT-Picture [44]), might be sufficient for predicting semantic storage loss, in which case the calculation of the additional components of the algorithm would be unnecessary. Also, previous studies [21, 39] have used MMSE scores as an indirect measure of disease progression to predict the emergence of a semantic storage disorder in people with AD. Thus, some researchers might argue that tests which measure severity of overall cognitive deficits would perform just as well, such as the MMSE or the Montreal Cognitive Assessment (MoCA [61]), an alternative measure of cognitive ability. Therefore, we compared the predictive ability of SSL Scores for participants’ ATL hypometabolism levels to that provided by its individual scores, as well as scores obtained from three other tasks: the MMSE, the MoCA, and CCT-Picture task. Scores were correlated with participants’ ATL hypometabolism levels, with their predictive strength compared to omnibus SSL scores.

### Assessing the predictive value of semantic storage loss scores: Consistency with PPA sub-type conditions

The previous literature demonstrating semantic processing versus semantic storage deficits has involved group studies focused on individuals within different diagnostic groups where a particular deficit was predicted[7, 21–24, 41–43, 45–47]. While the goal of developing SSL scores is to advance research by quantifying the degree of semantic storage impairment of each participant within their diagnostic group, it is certainly possible to derive average SSL scores for each diagnostic group as a whole. We will therefore carry out group comparisons across our three groups of PPA participants, where the degree of semantic storage deficit is predicted to be high (svPPA), medium (lvPPA), and low (nfvPPA), and predicted scores will be largely consistent with the degree of concrete object representation loss expected for different PPA sub-types. Thus, we predict a high average SSL score for the nfvPPA group, a medium range score for the lvPPA group, and a low score for the svPPA group. To be clear, these scores alone would be insufficient for assigning an individual to a particular PPA sub-type; however, demonstrating that participants’ scores were consistent with the level expected for a particular PPA group would further demonstrate concurrent validity for our scoring system.

## Materials and methods

### Participants

The study was carried out at the Jewish General Hospital in Montreal and approved by the Research Ethics Committee of the hospital. Participants were to be enrolled from the hospital outpatient Memory clinic based on clinical inclusion criteria. All participants were to be assessed as competent to participate by their treating physician. They were to be screened and diagnosed clinically by one of us who is a clinical expert in cognitive neurology (HC). We sought individuals with neurodegenerative diseases determined by one of us (HC) to meet criteria either for atypical AD with PPA [62], or FTD [63] with a predominant aphasic presentation. Participants were required to meet criteria of Mesulam for Primary Progressive Aphasia [25] with insidious aphasia, which was initially the most salient impairment, and where the aphasia was gradually progressive [26].

All participants were then classified clinically for best fit into one of the three PPA subtypes according to the subgrouping scheme of Gorno-Tempini [27] for svPPA, nfvPPA, and lvPPA. It should be noted that while rigorous attention was paid to the core features of these syndromes (for example the core features of PPA-G and PPA-L established by Mesulam [25, 26]), the ancillary features were not rigorously required for classification into the subgroups, in order to avoid a preponderance of “unclassified” participants. The core criteria used for sub-grouping are displayed below in Table 1.

**Table 1:** Core criteria used for classifying PPA sub-types.

Non-Fluent Variant PPA*	Logopenic Variant PPA*	Semantic Variant PPA*
**At least one core feature must be present**	**Both of the following core features must be present**	**Both of the following core features must be present**
Agrammatism in language production	Impaired single-word retrieval in spontaneous speech and naming	Impaired confrontation naming
Effortful, halting speech with inconsistent speech sound errors and distortions (apraxia of speech)	Impaired repetition of sentences and phrases	Impaired single-word comprehension

*PPA sub-type criteria from Gorno-Tempini et al. [27].

In addition to the criteria outlined in Table 1, participants were required to demonstrate in an initial screening that they had both anomia and semantic memory impairment. Using the Cambridge Semantic Battery [44], anomia was operationalized as scoring below the normal cut-off on the Cambridge Naming Task, while semantic memory impairment was operationalized as scoring below the normal cut-off on the picture version of Camels and Cactus task [64]. In this task, participants are presented a series of pages where each page displays an image of an object or animal at the top of the page (e.g., nail) and asked which of four images displayed at the bottom of the page is most associated with the top image (e.g., hammer presented alongside an axe, a screwdriver, and a wrench). Because these tasks were originally developed outside Montreal, Quebec, Canada, we administered both the Cambridge Naming Task and the Camels and Cactus tasks to a group of 20 normal elderly participants in Montréal (age range 66–87, 15 females, 12 anglophones, 8 francophones) to determine the inclusion cut-off for normative performance on the Cambridge Naming Task, as well as Camels and Cactus. No differences were found between the two linguistics groups, which is perhaps unsurprising as all presented stimuli was visual. We did allow for dialect and regional differences (e.g., *truck* for *lorry*), as well as related alternatives when the specific target word was unclear from the image (e.g. we accepted both *crocodile* and *alligator* for *crocodile* as both were plausible answers based on the image). Our elderly normal controls obtained an overall mean of 62.81 (*SD* = 1.00) on the Cambridge Naming Task. Thus, we established a cut-off score for abnormal naming of below 60 on the Cambridge Naming Task because these scores were more than 2 standard deviations lower than the normed mean. For the picture CCT, elderly normal participants had an overall mean of 58.80 (*SD* = 2.48); thus, we chose 54 as a cut-off because a score less than 54 would be more than two standard deviations below the mean.

### Formulation and validating the SSL

As previously discussed, it was predicted that a person with a semantic storage deficit would be unable to name a presented concrete object, even when given a phonemic cue [7, 65, 66], would show a consistent comprehension deficit for the same item on word-to-picture matching [67–77], and their naming ability would be unrelated to the severity of any cognitive deficit. To operationalize these semantic deficits, (failing to respond to cues, consistency, lack of correlation with cognitive function), we administered two tasks from the Cambridge Semantic Battery [44]: the 64-Item Cambridge Naming Task, and the Cambridge Word-Picture Matching Task (WPMT). We also administered the MMSE.

The non-MMSE tasks (naming, cued naming, word-picture matching) allowed us to check for cue effects and consistency, while also comparing two tasks with different cognitive demands. Naming and cued naming were administered concurrently. In the naming task, participants were presented 64 images (8 semantic categories, 8 images per category) one-by-one and asked to provide the name of the image. Note that we focused on concrete imageable item concepts, for which there is a far greater literature and consistent evidence of loss in brain damage. When participants failed to spontaneously name an item (e.g. *ostrich*), the participant was given the first two phonemes (i.e. *(os)*) as a cue. Older studies of semantic loss by Warrington and Shallice [75, 78–80] often documented this preservation of superordinate and category label knowledge in individuals with semantic storage deficits. This corresponds to the tendency of our participants to erroneously produce superordinate category labels during the naming task. Previous studies noted improved naming when the cues supplied were phonological or phonemic, indicative of retained semantic stores [12, 65, 80, 81] Thus, while somewhat counter-intuitive, use of phonemic or phonological cues is more effective than use of semantic cueing in these patients, and is accepted as an indicator of semantic processing difficulties. Indeed, participants in piloting would often spontaneously provide semantic generalities about objects when unable to name them (e.g. “you can eat it”, “You wear it”, “it’s an animal”); thus, we also expected semantic cues would be generally unhelpful as participants already seemed aware of such generalities despite being unable to produce the object’s name. In contrast, and consistent with past studies, phonemic cues were predicted to partially activate in memory the name being sought by the participant. After the concurrent naming and cueing tasks, participants were administered the WPMT, where each page presented one of the items from the 64-item naming task, alongside 9 distractor images from the same semantic category (e.g., a dog surrounded by other domestic animals). For each page, the name of an animal or object was given, and participants were asked to point to the corresponding image. Crucially, we checked whether the items missed in the initial naming and cueing tasks were now correctly identified in the WPMT. Finally, participants were administered the MMSE. It took approximately 60 minutes to administer all tasks to participants.

For each participant, we would calculate two proportions: a) the proportion of missed items initially missed in the spontaneous naming task that were correctly named when given a letter or phonemic cue; and b) the proportion of missed items initially missed in the spontaneous naming task that were correctly identified in the Word-to-Picture Matching Task. In both cases, proportions were converted to scores ranging from 0 (all missed items remained un-retrieved) to 1 (all missed items were retrieved). For the final measure, we calculated the person’s naming score out of 64, and the person’s MMSE score out of 30, before placing them into the ratio between a person’s naming proportion score and their MMSE proportion score. Like the other two components of the SSL score, this score will increasingly approach 0 when the person’s MMSE proportion score is greater than their Naming proportion score, but 1 when the MMSE proportion score is worse than the naming proportion score, which suggests the deficit may be due to poor cognitive impairment. All three scores were then averaged to give each an equal weight in the final omnibus semantic storage loss (SSL) score. Lower scores approaching 0 reflect more concrete object loss from semantic memory.

### PET-FDG image acquisition

PET/CT studies were performed at the Jewish General Hospital (Montréal, Québec) on a hybrid PET/CT scanner (Discovery ST, General Electric Medical Systems, Waukesha, WI, USA), which combines a dedicated, full-ring PET scanner with a 16-slice spiral CT scanner. Participants fasted for at least 4 hours. Approximately 5.55 MBq/kg (0.15 mCi/kg) of 18F-FDG was injected intravenously. Sixty minutes following 18F-FDG injection, CT and PET images were consecutively acquired from the base of the skull to the vertex. For the CT scan portion of the study, the settings were: 140 kVp, 120 mA, a rotation time of 0.8 s, a table speed of 17 mm per gantry rotation, a pitch of 1.75:1, and a detector row configuration of 6 × 0.625 mm. For the PET portion of the study, a 3D acquisition was performed using a 128 x 128 matrix and images were acquired using a single static 20 min bed position. PET attenuation-corrected, PET nonattenuation-corrected, CT, and fused images were reconstructed in the transaxial, coronal, and sagittal planes with an ordered subset expectation maximization (OSEM) iterative algorithm which incorporated both decay and attenuation correction. Images were displayed in a Xeleris 2.1 (General Electric Medical Systems, Waukesha, WI, USA) workstation for review, and were analyzed in 3D-SSP Neurostat (Department of Radiology, University of Washington, Seattle, WA, USA).

### MRI image acquisition and preprocessing

T1 images were acquired using MP-RAGE pulse sequences on a Siemens Trio 3.0 Tesla scanner at the Unité de Neuroimagerie Fonctionnelle at the Centre de recherche de l’Institut universitaire de gériatrie de Montréal, and comprised 1.0 mm thick sagittal slices. Image preprocessing was executed and coordinated by an automated pipeline developed at the Montreal Neurological Institute, comprising the following stages: [1] registration of T1 images to the MNI symmetrical ICBM152 non-linear 6th generation template using a 12-parameter linear transformation, [2] MR intensity nonuniformity correction, and [3] tissue classification into gray matter, white matter, and cerebrospinal fluid tissue types.

### Metabolism comparisons

#### PET-FDG image preprocessing

The participant’s FDG volume was first aligned to their corresponding native T1 scan using a 6-parameter, rigid-body fit, and then transformed into the ICBM152 stereotactic space, thus co-registering the scan with the T1 in ICBM-space. The resultant stereotactic-space dynamic volume was then blurred with a 6-mm full-width at half maximum Gaussian filter in order to increase signal-to-noise and minimize the effects of random high-frequency spikes in the data. A cerebellar gray matter mask was then applied against the dynamic volume, permitting us to compute an average intensity value within the cerebellar gray matter, to be used as reference when computing the participant’s FDG ratios at each voxel.

#### Computing FDG ratios

Ratios were computed by dividing the intensity value at each voxel of the dynamic volume with a reference value obtained from within the cerebellar gray. The cerebellar gray was used as a reference tissue since it is unlikely to be significantly affected by the pathologies exhibited by our participants, and the relatively large volume of tissue that comprises the cerebellar gray should provide us with relatively stable estimates. Thus, a ratio value of 1.00 can be said to reflect the cerebellar gray (the reference tissue) with regard to FDG uptake; areas with ratios less than 1.00 indicate less FDG uptake (relative to the cerebellar gray), whereas ratios greater than 1.00 show areas with a greater degree of FDG uptake. To enable analysis and interpretation of the voxel-level FDG ratios at the gyral-level, participants’ brains were automatically labelled by nonlinearly warping a modified AAL-based label volume from ICBM152-space onto each participant’s T1 (also in ICBM152-space). When applied to the PET-FDG volume, the participant-specific labels could be used to compute a mean FDG ratio at each of 120 regions of interest (ROIs). These regions of interest were consistent with automated anatomical labeling (AAL) based on the Colin27 brain.

## Results

### Anomic participants identified and classified clinically

We initially screened 32 individuals with NDD and anomia, but we excluded four participants because they had very mild anomia and a spontaneous naming score greater than 59. The remaining 28 participants all scored below the naming cut-off score on the Cambridge Naming Task, and were then classified clinically according to the *core* criteria of Gorno-Tempini [27] into the three predominant PPA subgroups *even if ancillary features were not present*. Twelve participants were classified as having a clinical diagnosis of lvPPA. For all of these individuals, the clinician strongly suspected (based on clinical assessment and imaging) that the underlying pathology was AD. The remaining 16 participants received a clinical diagnosis of FTD, aphasic variant. Of these individuals, 11 were classified as nfvPPA according to the core criteria of Gorno-Tempini [27]. They all met criteria for PPA-G according to the scheme of Mesulam. Five were diagnosed as having svPPA without behavioural problems.

Therefore, we accrued 28 individuals who all clinically met criteria for PPA and met the core criteria of the three categories of svPPA, nfvPPA, and lvPPA. We note that our nfvPPA participants in fact failed to satisfy all the ancillary criteria of Gorno-Tempini [27] in that there was neither completely spared object knowledge, nor completely spared content word comprehension on close testing. Similarly, our lvPPA group did not have completely spared object knowledge or completely spared single word comprehension. In this sense, the classification system of Mesulam was more closely adhered to [17]. Although others might term them PPA mixed or unclassifiable, we classified these participants as nfvPPA even when ancillary features were not rigorously adhered to. We note that Mesulam and others have chosen to consider about 20% of PPA cases as unclassifiable [82]. The lvPPA participants similarly met core criteria but not all ancillary criteria of Gorno-Tempini [27] in that there was neither completely spared object knowledge or completely spared single word comprehension on close testing. Thus, all participants met criteria for anomia and semantic deficits necessary for this study. This is consistent with a study by Galton [18] who found that 5 of 6 AD individuals that presented a lvPPA picture, in fact demonstrated semantic problems on in depth testing. Giannini referred to such participants as lvPPA + [19]. Similarly, Leyton [20] noted that 10% of nfvPPA and 20% of lvPPA (by strict criteria) still showed mildly impaired single word comprehension. Table 2 presents the demographic and neuropsychological information for these 28 participants. We present participants’ scores for Naming, Cued Naming, WPMT, MMSE, as well as their individual SSL scores. Groups were similar for age and years of education, and predominantly male. We note the likeliest designation of underlying brain pathology, explained in the text.

**Table 2:** Naming and neuropsychology data for PPA participants.

Participant	PPA Type	Likeliest Underlying Pathology	Sex	Age	Years of Education	Naming (max = 64)	Cued Naming (max = 64)	WPMT (max = 64)	MMSE (max = 30)	SSL (0.0–1.0)
KD	Non-Fluent	FTD	F	61	8	28	51	56	18	.61
MR	Non-Fluent	FTD	M	68	12	51	61	63	19	.75
BJ	Non-Fluent	FTD	M	72	15	57	64	64	28	.83
LR	Non-Fluent	FTD	M	73	12	40	56	62	6	.78
MA	Non-Fluent	FTD	F	63	18	56	59	64	26	.63
RP	Non-Fluent	FTD	M	59	14	26	50	52	17	.58
PS	Non-Fluent	FTD	M	73	12	57	63	62	22	.71
DL	Non-Fluent	FTD	M	68	13	15	33	38	8	.53
AS	Non-Fluent	FTD	F	71	16	46	59	64	23	.74
DC	Non-Fluent	FTD	M	75	11	58	63	64	27	.78
LS	Non-Fluent	FTD	F	67	16	53	61	60	12	.68
			Average	68.18	13.36	44.27	56.36	59.00	18.73	0.69
			SD	5.29	2.80	14.99	9.05	7.96	7.50	0.09
MC	Semantic	FTD	M	73	12	12	16	24	17	.19
WP	Semantic	FTD	M	82	12	7	21	38	10	.35
BF	Semantic	FTD	M	54	16	8	10	32	26	.20
ZD	Semantic	FTD	M	63	11	15	23	51	18	.39
TL	Semantic	FTD	F	71	18	14	17	49	26	.32
			Average	68.60	13.80	11.20	17.40	38.80	19.40	0.29
			SD	10.60	3.03	3.56	5.03	11.39	6.77	0.09
GD	Logopenic	AD	M	78	7	51	59	61	19	.65
OL	Logopenic	AD	M	65	11	30	40	57	23	.49
LA	Logopenic	AD	F	56	18	11	11	45	7	.36
FA	Logopenic	AD	F	77	16	10	15	40	8	.34
MG	Logopenic	AD	M	79	10	31	49	45	23	.45
CG	Logopenic	AD	M	78	9	21	34	58	12	.54
CC	Logopenic	AD	F	78	12	17	36	46	12	.47
MD	Logopenic	AD	M	64	11	30	50	53	14	.59
SC	Logopenic	AD	M	80	14	27	51	43	17	.50
WB	Logopenic	AD	F	60	18	3	20	26	2	.41
AM	Logopenic	AD	M	60	18	44	59	60	13	.72
BM	Logopenic	AD	F	72	18	12	36	54	10	.54
			Average	70.58	13.50	23.92	38.33	49.00	13.33	0.51
			SD	8.94	4.01	14.36	16.31	10.17	6.36	0.11

AD, Alzheimer’s Disease; F = Female; FTD, Fronto-Temporal Dementia; WPMT, Word-Picture Matching Task (Adlam et al., 2010); M = Male; MMSE, Mini-Mental State Examination (Folstein et al., 1975); SSL, Semantic Storage Loss; SD, standard deviation

All participants were further investigated with an FDG-PET scan, as well as a structural MRI.

### Imaging results

**Fig 2:**
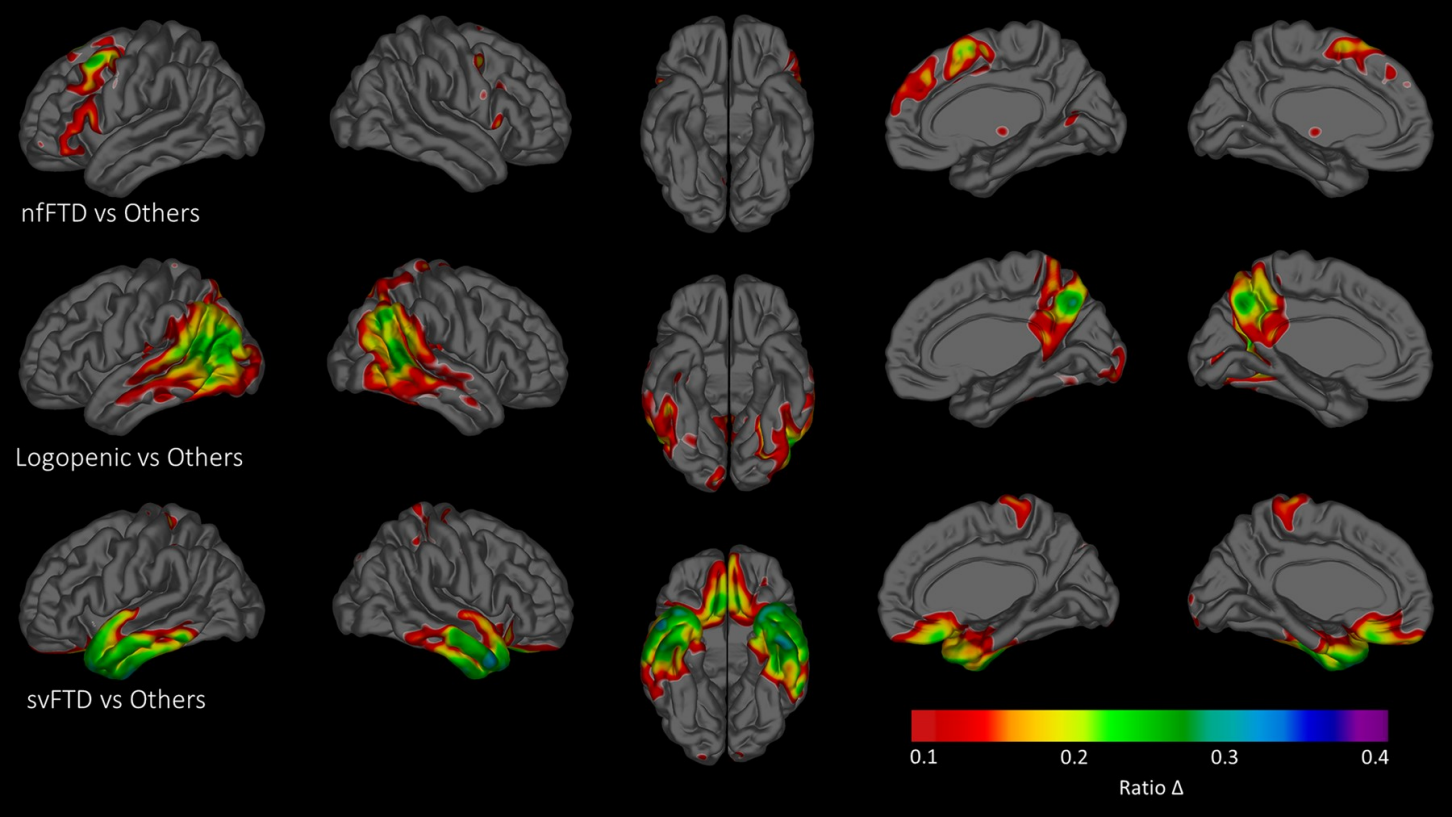
FDG PET results for the three PPA participant groups. Differences in cerebellar gray-relative FDG ratios, calculated per vertex, superimposed onto an averaged, elderly cortical surface. Images are thresholded such that only ratio differences greater than 0.1 are mapped to the spectral color bar. (Top row) Areas in which the nfvPPA group demonstrated a larger magnitude FDG ratio, when compared to the average of the svPPA and lfPPA groups. (Middle row) Comparison of lvPPA group FDG ratios against the averaged svPPA and nfvPPA groups, and (Bottom row) comparison of svPPA group ratios against the averaged nfvPPA and lvPPA groups.

FDG PET was carried out on all 28 individuals. For technical reasons, the right and left temporal lobes were visible in 27/28 participants. For assessment of group differences, we assessed average FDG ratios in 120 regions of interest. These were averaged in the 5 svPPA, 11 nfvPPA, and 12 lvPPA participants as three groups. The images derived are shown as Fig 2.

Displayed as a group, it is clear that the regions of hypometabolism correspond to the predicted anatomy of these syndromes. The nfvPPA participants demonstrate as a group marked hypometabolism in bilateral frontal regions, more on the left than the right. There is only mild hypometabolism extending to the anterior frontal region. The svPPA participants show marked hypometabolism of bilateral ATL regions, greater on the left than the right. The lvPPA participants as a group demonstrate hypometabolism in a more extensive region, involving right and left temporo-parietal regions [greater on the left] but also mild hypometabolism in bilateral frontal regions as well as the posterior cingulate. Within the temporal lobes, there is moderate hypometabolism that extends to the ATL, particularly noted on the left side.

### Semantic storage loss calculation and associated group membership

**Fig 3:**
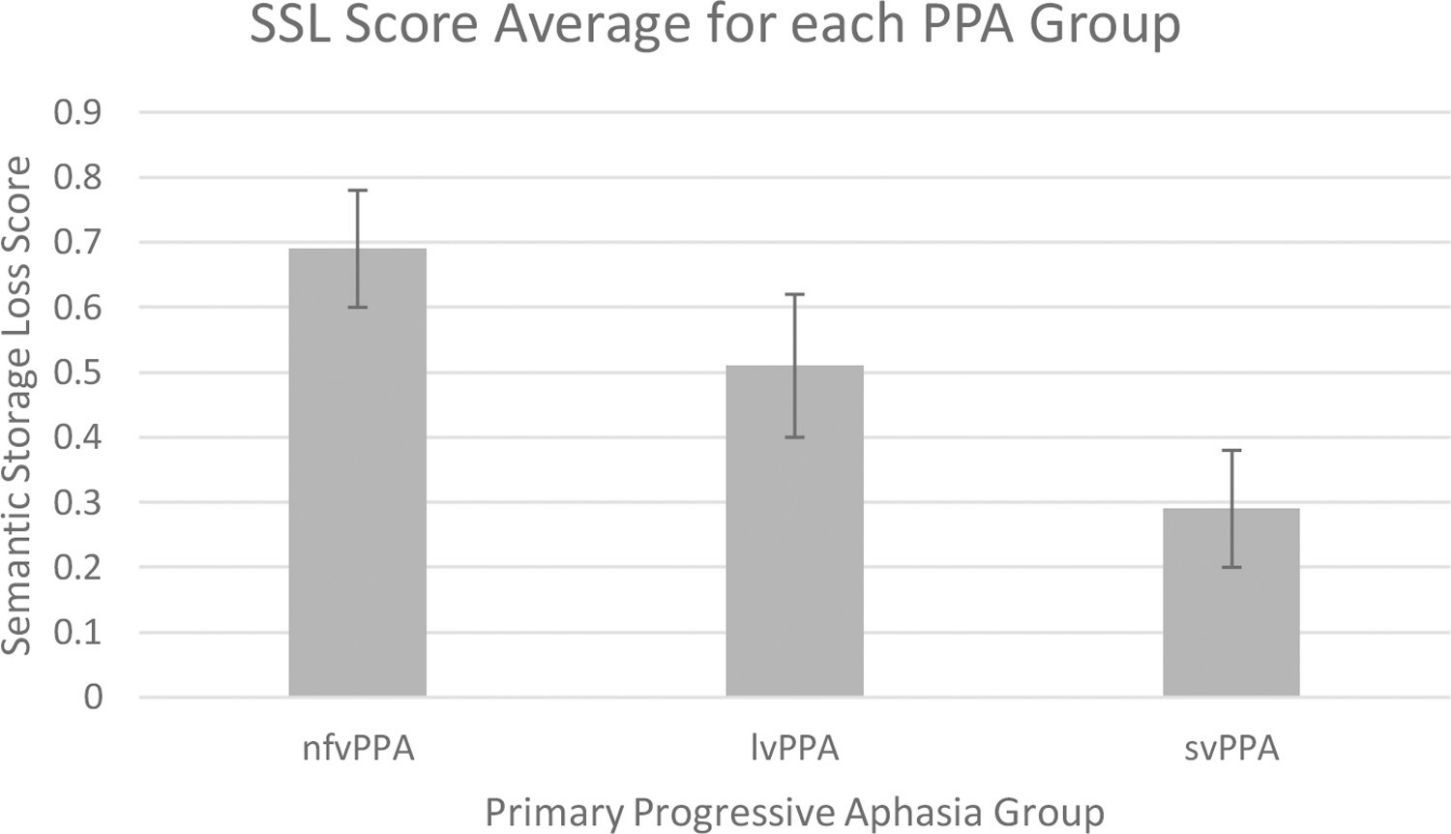
Bar graphs comparing the average semantic storage loss score found for each PPA group. Consistent with the prediction that semantic storage loss scores would be indicative of temporal, rather than frontal lobe damage, those participants in the svPPA group had the lowest SSL scores.

All statistics in this section and the following sections were performed using SPSS. The nfvPPA group (*n =* 11) had a high SSL average of 0.69 (*s*.*d*. = 0.09), while the svPPA group (*n* = 5) had a low SSL average of 0.29 (*s*.*d*. *=* 0.09). Meanwhile, the average SSL score for lvPPA participants (*n =* 12) was between the other two groups: 0.51 (*s*.*d*. *=* 0.11). Comparing these scores using a one-way anova, the omnibus test was significant (*F* (2, 25) = 26.90, *p* < .05), as well as post-hoc scheffe tests comparing the groups to each other. Thus, the results suggest that SSL scores were different across PPA sub-type. We also performed a one-way anova comparing MMSE and MoCA scores across groups, as well as age and years of education, but all results were non-significant. The SSL scores for the different groups are consistent with the rankings expected for the different PPA sub-type groups based on previous research. Therefore, SSL scores could serve as a helpful measure when forming an initial impression of a participant’s PPA sub-type; although the scores themselves are non-diagnostic nor make any conclusions beyond the degree of semantic storage loss. The average scores for each participants group are presented in Fig 3.

### Predicting hypometabolism levels from SSL scores

**Fig 4:**
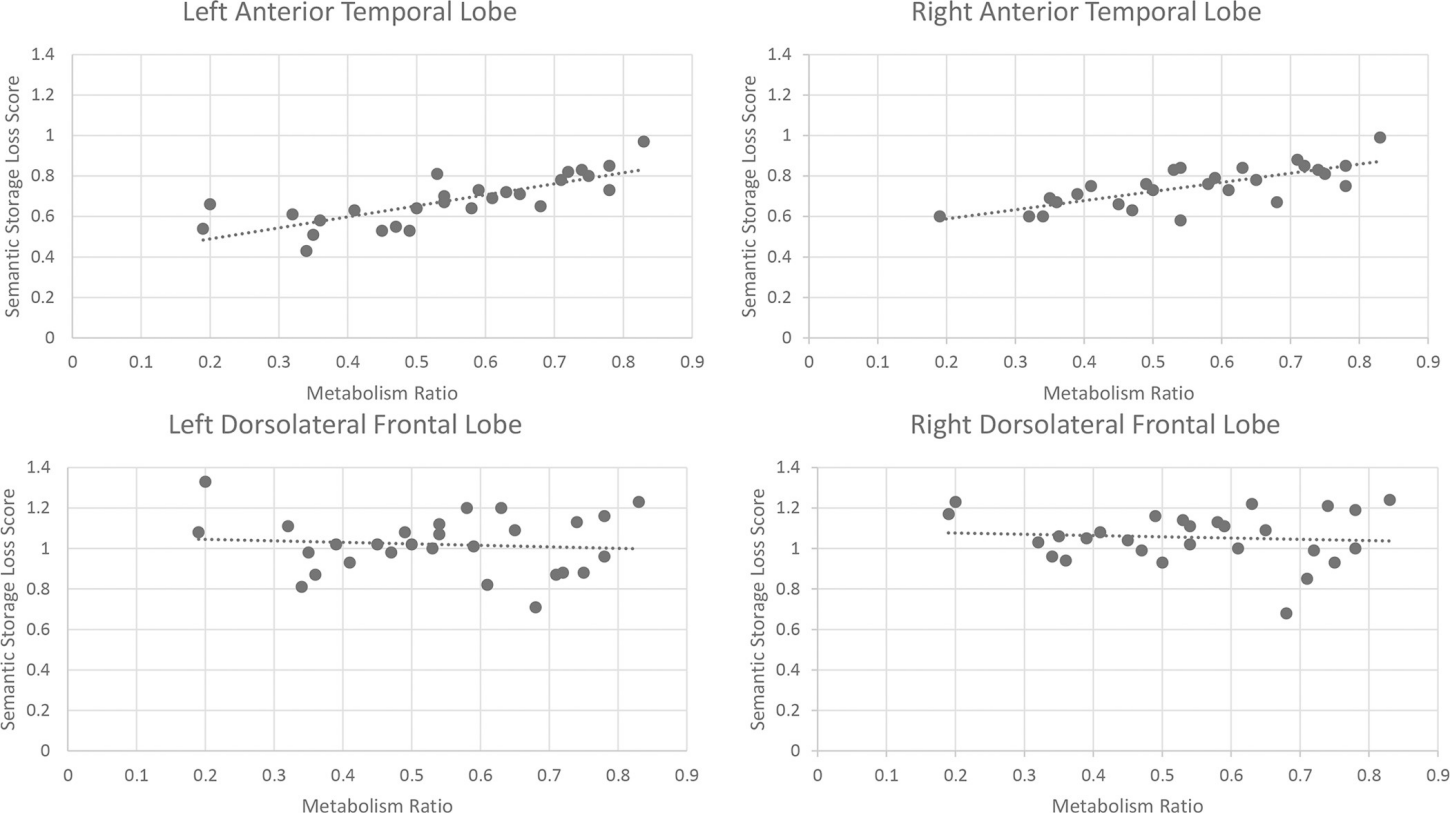
Scatterplots display the relationship found between participants’ semantic loss scores and their found metabolism ratio in different brain areas. Although semantic store loss scores are poor predictors of dorsolateral frontal lobe metabolism levels, they predicted metabolism levels in the left and right ATL.

As groups of people with svPPA have been shown to have greater ATL hypometabolism than groups of people with nfvPPA, it was hypothesized that our SSL scores would serve as strong predictors of ATL hypometabolism at an individual level. To test this prediction, we correlated the SSL score of all participants with the degree of hypometabolism in their left and right temporal pole. We were unable to obtain the metabolism level in the left temporal pole for one participant, nor the metabolism level in the right temporal pole of another participant. Thus, each correlation was run for 27 participants. We found a significant correlation between SSL scores and left ATL hypometabolism (*r* (27) = .78, *p* < .001), as well as a significant correlation between SSL scores and right ATL hypometabolism (*r* (27) = .73, *p* < .001). When we correlated SSL scores with the hypometabolism of the left and right dlpFC, no significant correlations were found. In contrast, participants’s’ MoCA and MMSE scores did correlate with hypometabolism in the dlpFC (*MoCA*, left dlpFC, *r* (28) = .60, *p* < .01, right dlpFC, *r* (28) = .44, *p* < .01; *MMSE*, left dlpFC, *r(28) =* .*58*, *p* < .01, right dlpFC r(28) = .42, *p* < .05). Fig 4 below displays the SSL score correlations. Participants’ MoCA and MMSE scores did correlate (*r* (28) = .89, *p* < .001).

Having established that SSL scores correlate with ATL hypometabolism, we next checked if the SSL scores would correlate with ATL hypometabolism when the analysis was restricted to participants living with lvPPA. Recall that our interest in this analysis comes from the argument that greater levels of hypometabolism in the ATL should coincide with the emergence of a semantic storage deficit in people living with AD. We therefore carried out a similar analysis, utilizing only data from the 12 lvPPA participants. For both the left and right ATL, there was a significant correlation with SSL scores, albeit more strongly for the left ATL (*left* (*r* (12) = .85, *p* < .01), *right* (r (12) = .62, *p* < .05). Thus, SSL scores could help in exploring when semantic storage deficits emerge in AD.

Finally, as a post-hoc analysis, we examined if any of the individual components of the SSL score (i.e., the ratio between the MMSE and Naming score; proportion of unnamed items correctly named when given a cue; proportion of unnamed items correctly recognized when presented in WPMT) would correlate with ATL hypometabolism better than the omnibus SSL score. We also examined other combinations: the average of the proportion of unnamed items correctly named when given a cue and the proportion of unnamed items correctly recognized when presented in WPMT; once without participants naming proportion score and once with their naming proportion score. Finally, we ran correlations with other assessment scores that some researchers may consider predictive: MMSE, Cambridge Naming Score, CCT-Picture, and the MoCA. For example, participants’ CCT-Picture and SSL scores had a positive correlation (*r* (28) = .59, *p* < .01); thus, it was worth examining which set of scores correlated with ATL hypometabolism.

Compared to SSL Scores, which correlated positively with left and right ATL hypometabolism both when all participants, or only participants with lvPPA were considered, all other measures, with the exception of the MMSE to Naming Ratio component of the SSL, failed to reach significance for one of the examined correlations. Alternatively, because the MMSE to Naming ratio, like the SSL scores, was found to correlate with ALT hypometabolism, both when all participants and when only lvPPA participants were considered, it is possible the MMSE to Naming ratio is sufficient, and it is the other sub-components that are unneeded. To confirm that SSL scores were a better predictor of hypometabolism than the MMSE to naming ratio alone, we ran a hierarchical regression to compare two models. For both models, left and right ATL hypometabolism was the dependent variable. In model 1, the only predictor was the score representing the ratio between the MMSE score and the Naming score, whereas model 2 also included the full SSL score to observe if the full SSL score would show a significant improvement in R^2^ (the proportion of variance explained in the DV by the model). Thus, this is a framework for model comparison rather than a statistical model; the first model had the ratio value only, while the second model also had the omnibus SSL score to observe if the R-Square Change Statistic would be significant. When the only predictor was the MMSE to naming ratio score, the anova was significant for both ATLs (all participants: *Left ATL*, *R*^*2*^ = .24, *F* (1,26) = 7.9, *p* < .001; *Right ATL*, *R*^*2*^ = .26, *F* (1,26) = 8.6, *p* < .001), but *R*^*2*^ was larger when SSL scores were added (left ATL ΔR^2^ = 0.41; right ATL ΔR^2^ = 0.29), and produced significant R-Square Change Statistics (all participants: *Left ATL*, *R*^*2*^ = .65, *F* (2,26) = 22.12, *p* < .001, *R*^*2*^ change = .41, *F* (1,24) = 27.85, *p* < .001; *Right ATL*, *R*^*2*^ = .54, *F* (2,26) = 14.28, *p* < .001, *R*^*2*^ change = .29, *F* (1,24) = 15.15, *p* < .01). Therefore, the best general predictor of left and right ATL hypometabolism, regardless of PPA sub-type, was the SSL scores. Correlations are presented in Table 3 below.

**Table 3:** Predicting ATL hypometabolism in PPA participants.

	LEFT ATL (All Participants)	RIGHT ATL (All Participants)	LEFT ATL (PPAlogo Participants)	Right ATL (PPAlogo Participants)
SSL Score	*r =* 0.78 *p* < .001	*r =* 0.73 *p* < .001	*r =* 0.85 *p* < .001	*r =* 0.62 *p* < .05
MMSE to Naming Ratio	*r =* 0.49 *p* < .01	*r =* 0.51 *p* < .01	*r =* 0.72 *p* < .01	*r =* 0.73 *p* < .01
Average of: % of missed items recalled with Cues and % of missed items recognized in WPMT	*r =* 0.80 *p* < .001	*r =* 0.73 *p* < .001	*r =* 0.76 *p* < .01	Non-Significant
Average of: % of missed items recalled with Cues, % of missed items recognized in WPMT, and Naming Score (Score obtained /64)	*r =* 0.78 *p* < .001	*r =* 0.73 *p* < .001	*r =* 0.68 *p* < .05	Non-Significant
Cambridge Naming Score	*r* = 0.67 *p* < .05	*r* = 0.66 *p* < .05	Non-Significant	Non-Significant
% of missed items recalled with Cues	*r =* 0.76 *p* < .001	*r =* 0.68 *p* < .001	*r =* 0.65 *p* < .05	Non-Significant
% of missed items recognized in WPMT	*r =* 0.62 *p* < .01	*r =* 0.55 *p* < .01	Non-Significant	Non-Significant
CCT Picture Score	*r =* 0.45 *p* < .05	*r =* 0.51 *p* < .001	Non-Significant	Non-Significant
MoCA	*r =* 0.43 *p* < .05	*r =* 0.43 *p* < .05	Non-Significant	Non-Significant
MMSE	Non-Significant	Non-Significant	Non-Significant	Non-Significant

## Discussion

Our primary goal was the production of an algorithm that would quantify the degree of semantic storage loss in a person with PPA. Crucially, we aimed to ascertain and measure the degree of semantic storage loss rather than simply identifying if semantic memory was normal or impaired, as has been carried out previously using measures like the Camels and Cactus task. We established a scoring system *a priori* for our participants based on their performance on four tasks: MMSE, spontaneous naming, cued naming, and WPMT. We refer to the average of these scores as semantic storage loss scores (SSL scores) as they are designed to predict the degree of a participant’s concrete object representation loss from semantic memory; assumed to coincide with ATL damage, and present behaviorally as a semantic storage deficit. We ran several analyses to validate our scoring system. First, we verified that our SSL scores strongly correlated with left and right ATL hypometabolism in participants with variant forms of PPA, supporting the prediction that lower scores, reflecting semantic storage impairment, would also be indicative of greater temporal pole hypometabolism. Results were consistent with this prediction, and in addition, these same scores were non-predictive of the degree of dlpFC hypometabolism; thus, SSL scores’ predictive value appears specific for ATL hypometabolism.

When we compared the SSL scores for three PPA sub-type groups, we assumed that these three groups would exhibit different levels of semantic storage impairment (low level in nfvPPA, medium level in lvPPA, high in svPPA). To be clear, SSL scores are insufficient as the only diagnostic criteria for differentiating these three sub-types. Instead, we sought to demonstrate the ability of the SSL score to demonstrate group differences in the degree of a semantic storage deficit consistent with predictions for the different PPA variants. More specifically, we expected the comparison of PPA sub-types to demonstrate a spectrum where the degree of concrete object representation loss across PPA sub-types would represent two distinct extremes: near capacity in nfvPPA and nearly empty in svPPA. Consistent with this prediction, the nfvPPA group had scores closer to 1, while the svPPA groups had scores closer to 0. Between both groups, lvPPA participants were at the mid-point (0.5), suggesting some degree of concrete object representation loss, but less severe than that observed in svPPA, where representation loss is expected to be more severe.

Next, we examined if this correlation would hold even if only participants with lvPPA were examined, as it has been argued that semantic storage deficits in AD can emerge with increasing severity. We found SSL scores correlated strongly with ATL hypometabolism in a group of lvPPA individuals. Indeed, in our final set of correlations, we demonstrated that the omnibus SSL score correlated better with ATL hypometabolism that did MMSE scores, as well as other possible tasks (e.g. MoCA), including those designed to examine impaired semantic memory (e.g., CCT-Picture), and verified that the average of the individual components was a stronger predictor of ATL hypometabolism than just the SSL ratio score. Curiously, the importance or value of the different sub-components seemed to change if all participants were considered versus just those with lvPPA. The proportion of items recalled when given a cue, especially if averaged with the proportion of items initially unnamed but then identified on the WPMT, produced correlations as strong as the SSL score itself. Therefore, the average of these two items may serve as a general indicator of semantic storage loss when different variants are considered, but it appears to lack a sensitivity that is gained by the addition of the MMSE to Naming Ratio. When only lvPPA participants were examined, it was the MMSE to Naming Ratio that correlated with ATL hypometabolism. Therefore, the inclusion of this sub-component may be superfluous for detecting gross differences, but the MMSE to Naming Ratio gives the SSL an extra level of precision because it seeks to incorporate to what extent performance may have been impacted by general cognition. In other words, it seeks to integrate the possibility that a participant’s’ performance was affected by their general cognition as opposed to semantic storage loss. This factor may be critical for lvPPA, where poor general cognition is possible [83].

Indeed, for lvPPA participants, the Naming to MMSE ratio was the sub-component that best correlated with levels of ATL hypometabolism. There are two possibilities that can explain this result. First, it is possible that the added sensitivity provided by the Naming to MMSE ratio is lost when groups expected to have gross distinctions in terms of semantic storage loss are considered. This added sensitivity, however, allows for SSL scores to significantly correlate with found ATL hypometabolism levels even when a smaller range of hypometabolism levels are considered. Alternatively, the need to incorporate a participant’s level of general cognition may be especially important for lvPPA participants. As previously discussed, as the disease progresses for people with lvPPA, it is possible for such participants to develop symptoms similar to svPPA due to progressive damage of the ATL [84–85]. Similarly, some researchers have made a distinction between those traditionally labelled as lvPPA and a sub-set of lvPPA participants who display semantic difficulty and word comprehension difficulties. Consequently, for lvPPA, it may be especially important to distinguish to what degree their performance on the given tasks is related to general cognition or ATL damage.

### Limitations

The present algorithm represents an initial foray into quantifying the degree of semantic storage loss in an individual. Presently, it has the potential to serve as an identifier of svPPA, but we caution its use as a diagnostic tool. This potential reflects svPPA itself being associated with semantic storage loss and ATL hypometabolism, which the SSL is designed to measure and expected to be present in people with svPPA. Therefore, it is perhaps unsurprising that the individual components of the SSL correlate with the symptom profile of svPPA, For this same reason, however, it can have difficulty distinguishing those PPA variants where semantic processing, rather than storage, are more central symptoms (i.e. nfvPPA and lvPPA). Furthermore, it makes no prediction about the different profiles expected for people with nfvPPA and lvPPA. These PPA variants have symptoms unassociated with semantic storage loss (e.g. agrammatism), which are not measured or accounted for by the SSL. In summary, the SSL might be good at detecting svPPA, but its ability to separate the other PPA variants is problematic.

Further refinement and additional studies will also be needed to verify its applicability for different languages and cultures, as well as diagnoses other than PPA such as stroke. While norms based on age, sex, and education levels are also possible, we must stress that the SSL’s goal isn’t the identification of semantic memory deficits, but rather to measure the degree of semantic storage loss. In other words, the SSL is probably best used when anomia symptoms are already present to ascertain the degree of severity. Future versions of the SSL may also use a task other than the MMSE to assess the level of cognitive impairment. More specifically, it might be advantageous to use a task believed to measure only the degree of cognitive impairment in an individual with the absence of any confounds or alternative explanations for the score obtained on the task. Finally, it must be noted that the SSL is primarily designed to explain anomia in reference to Naming, and only then in reference to concrete objects. Semantics related to verbs, abstract nouns, or abstractions, are left entirely unexamined and are beyond the scope of the SSL. Thus, SSL scores are limited as an index of concrete object representation rather than verbs or abstract nouns.

### Conclusion

SSL scores can be utilized to assess the degree of concrete object representation loss in an individual. The scores, in turn, correlate with the degree of related ATL damage. The quantitative information provided by a score on a continuum from 0.0 to 1.0 allows for a sensitive reading of the person’s impairment.
